# Books and Pamphlets Received

**Published:** 1885-12-20

**Authors:** 


### BOOKS AND PAMPHLETS RECEIVED.

*A Reference Hand-book of the Medical Sciences;* being a complete and convenient book of reference for information upon topics belonging to the entire range of scientific and practical Medicine, and consisting of a series of concise essays, and brief paragraphs, arranged in the alphabetical order of the topics of which they treat, prepared by writers who are experts in their respective departments. Illustrated by chromo-lithographs and fine wood engravings. Edited by Albert H. Buck, M. D., New York City. Volume I. New York: William Wood & Co., 56 and 58 LaFayette Square. 1885.

In undertaking to give a general view of the scope and character of this work it seems most becoming to present the prefatory remarks of the editor, indicating that it is essentially a collection of articles—some brief, others of considerable length—treating of many of the more important topics in regard to which medical men are likely to desire information. He has been guided in the selection of topics by the size of the entire work, it being in eight volumes of about 800 pages each, so as to gain enough space for a fairly satisfactory and thorough treatment of, at least, the more important subjects. While he has given a large share of this space to matters of a practical nature—the diagnosis and treatment of disease—he has not forgotten to make ample provision for such departments as medical botany, climatology, embryology, physiological and pathological chemistry, applied anatomy, medical jurisprudence, military and naval surgery. In harmony with these considerations, he has concluded to leave out separate articles on some of the topics which appear as independent headings in other works of a similar character; and, on the other hand, he has introduced articles on many topics which are not discussed in any of the treaties which ordinarily find their way into the libraries of medical men. This volume is not self-indexing, so that much valuable space might be saved in it and the succeeding volumes, and with a view to introducing, instead, at the end of the work, a reasonably full index of all the different subjects. The alphabetic order of the topics will enable the reader to find the article that may be sought, in the respective volumes, upon the back of which the first and last syllables are printed, so as to indicate their contents · and there is a great advantage in having the index of the whole at the close of the last volume, thus facilitating the search for a special subject. The topics are generally found of a professional nature, and merely embrace an extensive and exceedingly interesting array of subjects, covering the entire domain of medical science, and treated in a practical manner, and brought down to the latest advances.

With such an opening for this bill of fare, comprising eight courses, the medical epicure has a favorable prospect of a most sumptuous feast in the completion of this great undertaking; and all the surroundings and embellishments encourage the belief that the most fastidious appetite will be satisfied. The artistic skill employed in the execution of the work fills the full measure of our expectations, based upon the former admirable productions of the energetic publishers; and we congratulate the student and practitioner upon the appearance of this first volume of the "Reference Hand-book of the Medical Sciences."

*A Treatise on Aseatic Cholera.* Edited and prepared by Edmund Charles Wendt, M. D., Curator and Pathologist of the Saint Francis Hospital; Curator and Pathologist of the New York Infant Asylum; Member of the New York Pathological Society, of the Neurological Society, of the Medical Society of the county of New York, etc.; in association with Drs. John C. Peters, of New York; Elz. McClellan, U. S. A.; John B. Hamilton, Surgeon-Genaral U. S. Marine Hospital Service, and George M. Sternberg, U. S. A. Illustrated with maps and engravings. New York: William Wood & Co., 56 LaFayette Place. 1885.

This book contains over 400 octavo pages, and its object "is to furnish the physician with a faithful account of the actual state of our knowledge" regarding cholera. The general history of the disease, and of the principal epidemics since 1835, is described by Dr. Peters. The early history of the cholera in India is also given, and the great epidemic of 1819 is graphically described. The several incursions in the United States is mentioned. The etiology of the disease is ably considered, with its symptomatology, morbid anatomy, pathological histology, diagnoses, treatment, prevention, etc.

*On Renal and Urinarg Affections.* By W. Howship Dickinson, M. D., Cantab. F. R. C. P., Physician to, and Lecturer on Medicine at, St. George's Hospital; Consulting Physician to the Hospital for Sick Children; Corresponding Member of the Academy of Medicine of New York. Miscellaneous affections of the Kidneys and Urine. New York: William Wood & Co. 1885.

This is an illustrated work of 337 octavo pages. It is clinical and practical. Among the many subjects considered, the pathology and varieties of renal tumors are fully treated; also tubercle and hydronephrosis and pyonephrosis. Cystic diseases and renal calculi are ably discussed, as also albuminuria in relation to other disorders.

*Letters from a Mother to a Mother* on the formation, growth, and care of Teeth. By the Wife of a Dentist, Mrs. M. W. J. Honoray, Member of the Southern Dental Association; Corresponding Editor of Archives of Dentistry, St. Louis, etc. Welch Dental Company, No. 1403 Filbert street, Philadelphia. 1885.

A valuable little work, of over one hundred pages, on a subject of interest and importance.

*The Oleates;* an investigation into their nature and action. By John V. Shoemaker, A. M., M. D., Lecturer on Dermatology at the Jefferson Medical College; Physician to the Philadelphia Hospital for Skin Diseases, etc. Philadelphia: F. A Davis, attorney, 1217 Filbert street. 1885.

*Household Receipts.* Valuable receipts for those who regard economy as well as excellence. Published by Joseph Burnett & Co., Boston. 69 pages. Price, 25 cents.

*A Study of Epilepsy;* a paper read before the American Medical Association. By J. J. Caldwell, M. D. Baltimore, Md.

